# Supplementary material for: Conjoined Genes as Common Events in Childhood Acute Lymphoblastic Leukemia
Source: Cancers (Basel). 2022 Jul 20;14(14):3523. doi: 10.3390/cancers14143523 (PMC9315513; doi:10.3390/cancers14143523)

Supplementary Figures S1-S27

CONJOINED GENES........ 2

SUPPLEMENTARY FIGURE S1. (AC019118.3::AC019118.2)........ 2
SUPPLEMENTARY FIGURE S2. (CACUL1::RP11-427L15.2)........ 2
SUPPLEMENTARY FIGURE S3. (CCPG1::PIGBOS1)........ 2
SUPPLEMENTARY FIGURE S4. (CTB-26E19.1::COL23A1)........ 3
SUPPLEMENTARY FIGURE S5. (FAM200B::BST1)........ 3
SUPPLEMENTARY FIGURE S6. (KLHL22::SCARF2)........ 4
SUPPLEMENTARY FIGURE S7. (MYNN::RP11-362K14.7)........ 4
SUPPLEMENTARY FIGURE S8. (PPP1R3F::LL0XNC01-7P3.1)........ 4
SUPPLEMENTARY FIGURE S9. (RP11-20D14.3::RIMKLB)........ 5
SUPPLEMENTARY FIGURE S10. (RP11-397H6.1::RP11-541G9.1)........ 6
SUPPLEMENTARY FIGURE S11. (RP11-87G24.3::RP11-87G24.6)........ 6
SUPPLEMENTARY FIGURE S12. (TMEM86A::RP11-1081L13.4)........ 7

FUSION TRANSCRIPTS........ 8

SUPPLEMENTARY FIGURE S13. (DCAF8::ZNF836)........ 8
SUPPLEMENTARY FIGURE S14. (DMD::STAMBPL1)........ 8
SUPPLEMENTARY FIGURE S15. (IK::FBXW2)........ 9
SUPPLEMENTARY FIGURE S16. (INPP5A::SETD7)........ 9
SUPPLEMENTARY FIGURE S17. (MAEA::CTBP1)........ 10
SUPPLEMENTARY FIGURE S18. (MAML2::FAT3)........ 10
SUPPLEMENTARY FIGURE S19. (MNT::CLUH)........ 11
SUPPLEMENTARY FIGURE S20. (NFX1::DICER1)........ 11
SUPPLEMENTARY FIGURE S21. (PAX5::POM121C)........ 12
SUPPLEMENTARY FIGURE S22. (RP11-148O21.2::ATG4B)........ 12
SUPPLEMENTARY FIGURE S23. (SLFNL1::SMPD2)........ 13
SUPPLEMENTARY FIGURE S24. (TMEM263::CD47)........ 13
SUPPLEMENTARY FIGURE S25. (TSKS::ARRDC2)........ 14
SUPPLEMENTARY FIGURE S26. (ZC3H12D::RP11-445F6.2)........ 14
SUPPLEMENTARY FIGURE S27. (ZNF444::HLA-B)........ 15

Conjoined genes

Supplementary Figure S1. (AC019118.3::AC019118.2)

Supporting reads for conjoined gene AC019118.3::AC019118.2. UCSC Genome Browser representation of the reads spanning over the two partners, as well as basic gene models and mRNA evidence are provided.

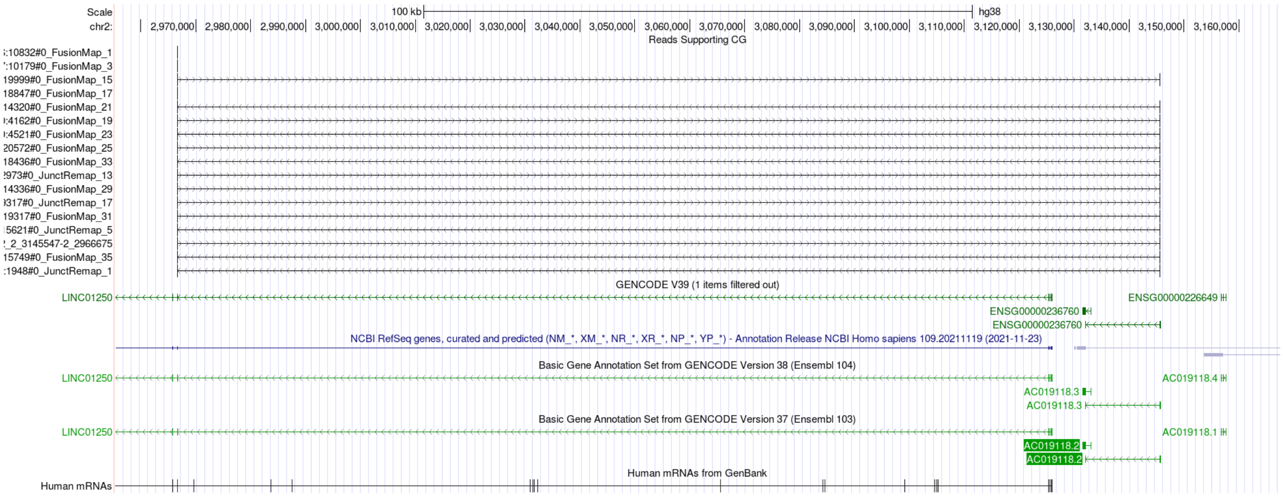

Supplementary Figure S2. (CACUL1::RP11-427L15.2)

Supporting reads for conjoined gene CACUL1::RP11-427L15.2. UCSC Genome Browser representation of the reads spanning over the two partners, as well as basic gene models and mRNA evidence are provided.

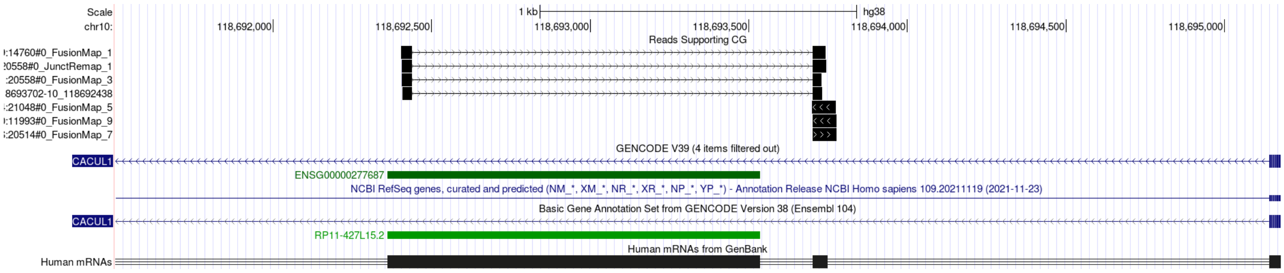

Supplementary Figure S3. (CCPG1::PIGBOS1)

Supporting reads for conjoined gene CCPG1::PIGBOS1. UCSC Genome Browser representation of the reads spanning over the two partners, as well as basic gene models and mRNA evidence are provided.

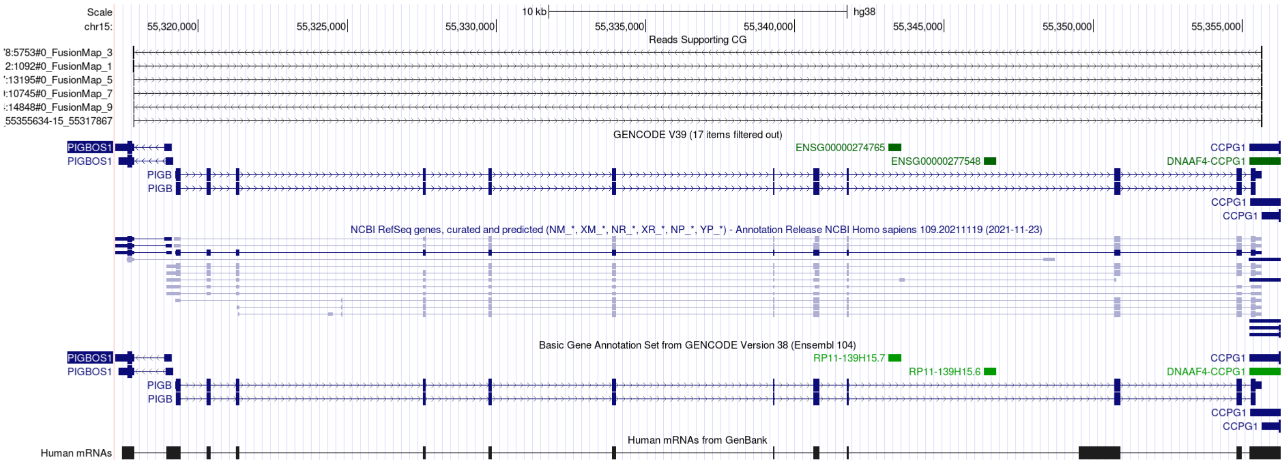

Supplementary Figure S4. (CTB-26E19.1::COL23A1)

Supporting reads for conjoined gene CTB-26E19.1::COL23A1. UCSC Genome Browser representation of the reads spanning over the two partners, as well as basic gene models and mRNA evidence are provided.

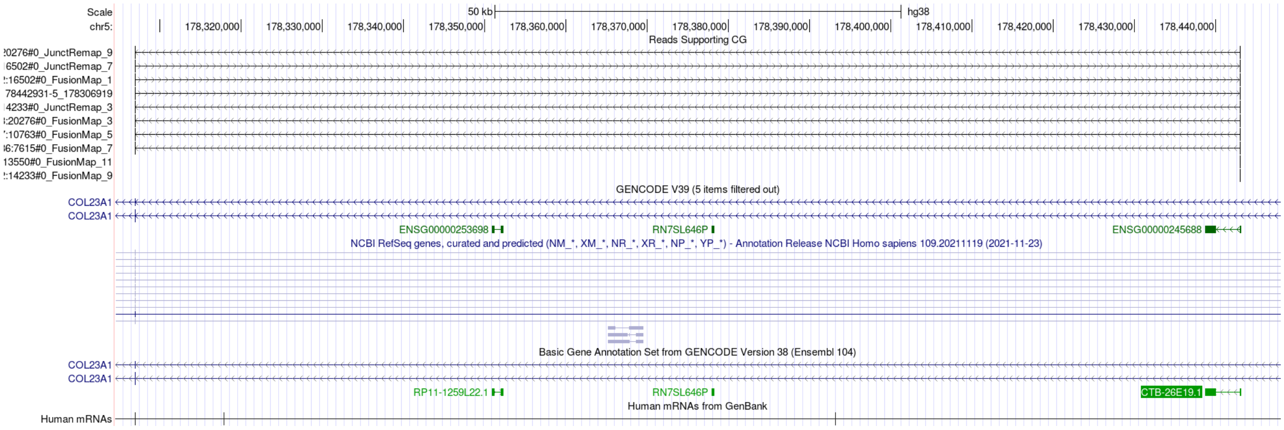

Supplementary Figure S5. (FAM200B::BST1)

Supporting reads for conjoined gene FAM200B::BST1. UCSC Genome Browser representation of the reads spanning over the two partners, as well as basic gene models and mRNA evidence are provided.

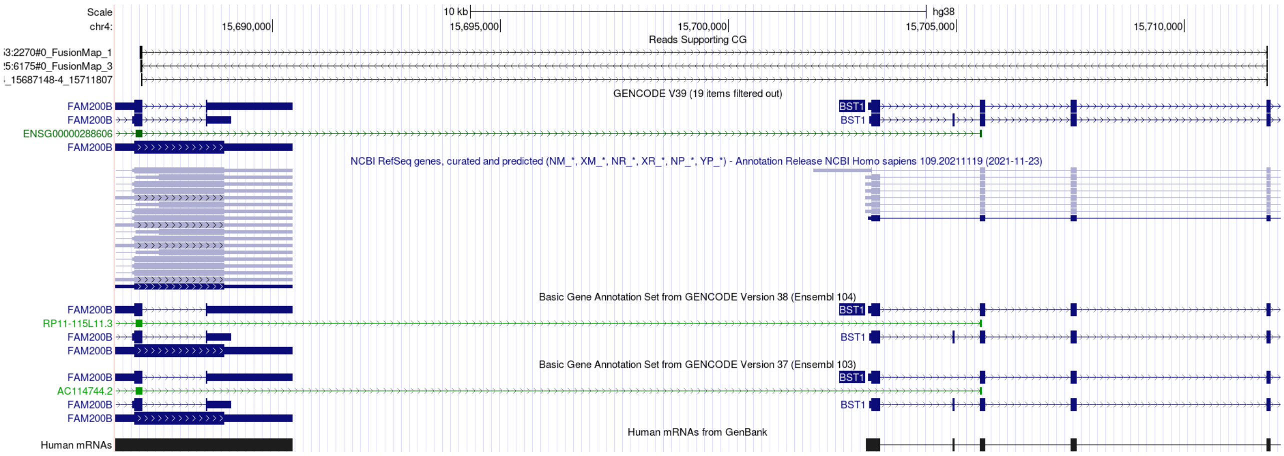

Supplementary Figure S6. (KLHL22::SCARF2)

Supporting reads for conjoined gene KLHL22::SCARF2. UCSC Genome Browser representation of the reads spanning over the two partners, as well as basic gene models and mRNA evidence are provided.

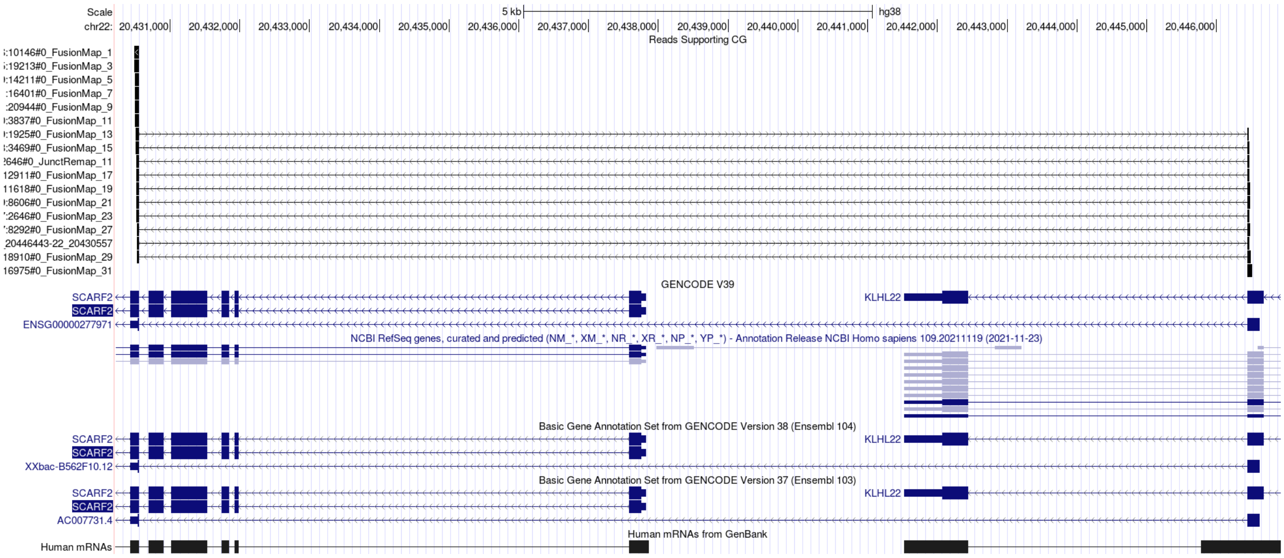

Supplementary Figure S7. (MYNN::RP11-362K14.7)

Supporting reads for conjoined gene MYNN::RP11-362K14.7. UCSC Genome Browser representation of the reads spanning over the two partners, as well as basic gene models and mRNA evidence are provided.

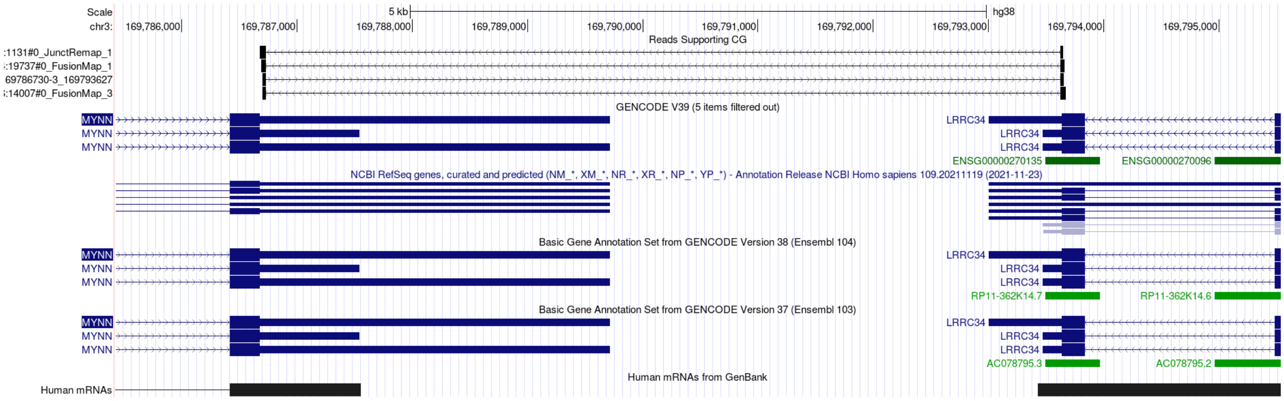

Supplementary Figure S8. (PPP1R3F::LL0XNC01-7P3.1)

Supporting reads for conjoined gene PPP1R3F::LL0XNC01-7P3.1. UCSC Genome Browser representation of the reads spanning over the two partners, as well as basic gene models and mRNA evidence are provided.

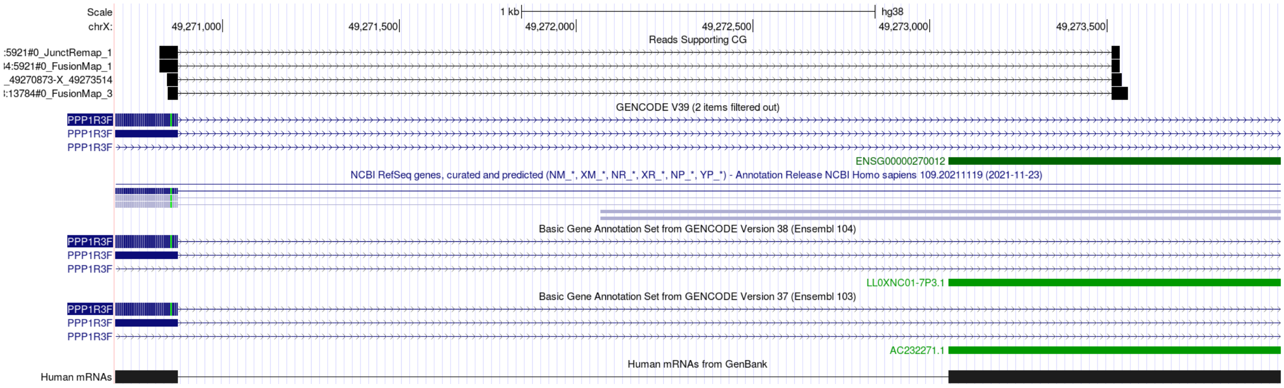

Supplementary Figure S9. (RP11-20D14.3::RIMKLB)

Supporting reads for conjoined gene RP11-20D14.3::RIMKLB. UCSC Genome Browser representation of the reads spanning over the two partners, as well as basic gene models and mRNA evidence are provided.

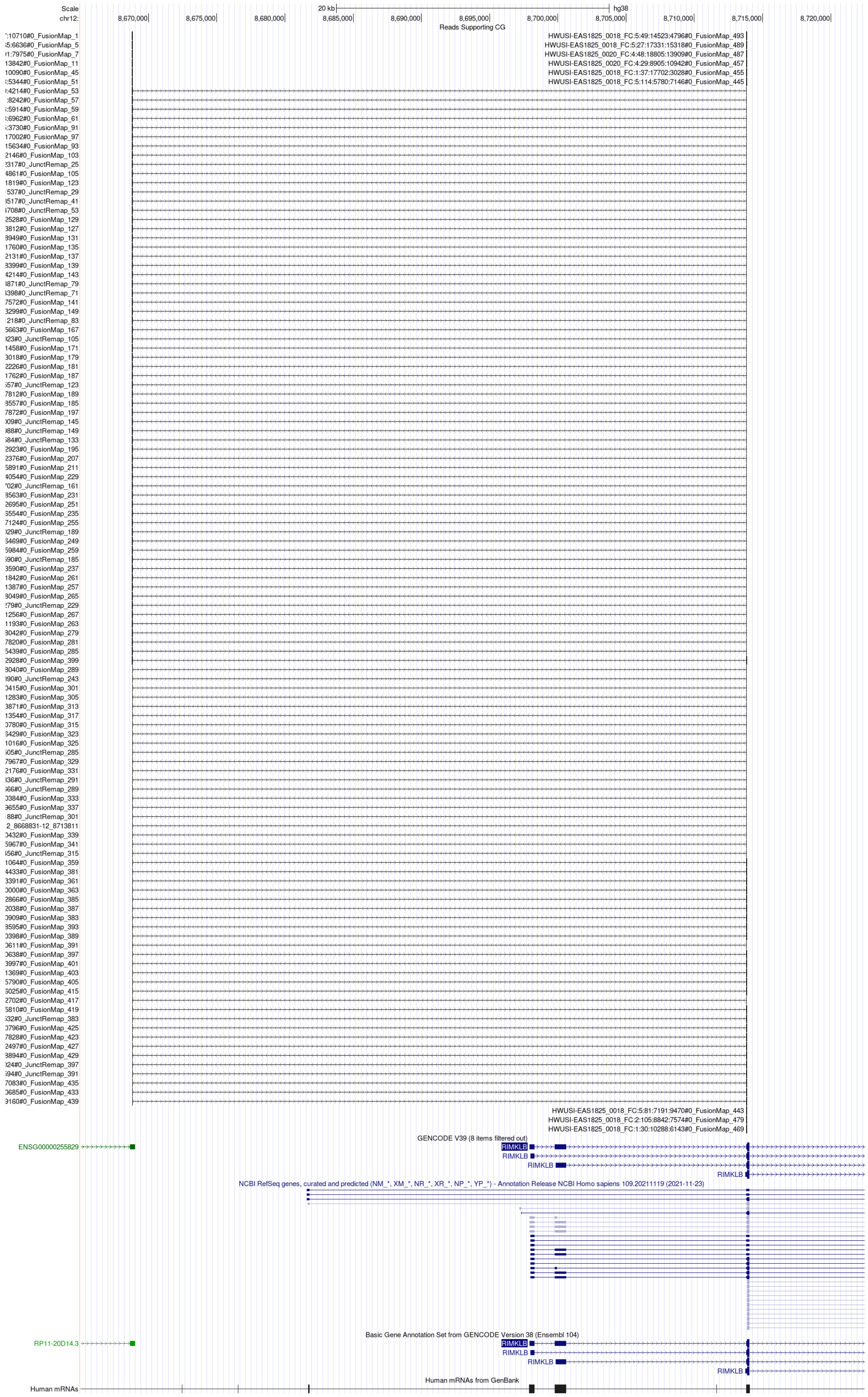

Supplementary Figure S10. (RP11-397H6.1::RP11-541G9.1)

Supporting reads for conjoined gene RP11-397H6.1::RP11-541G9.1. UCSC Genome Browser representation of the reads spanning over the two partners, as well as basic gene models and mRNA evidence are provided.

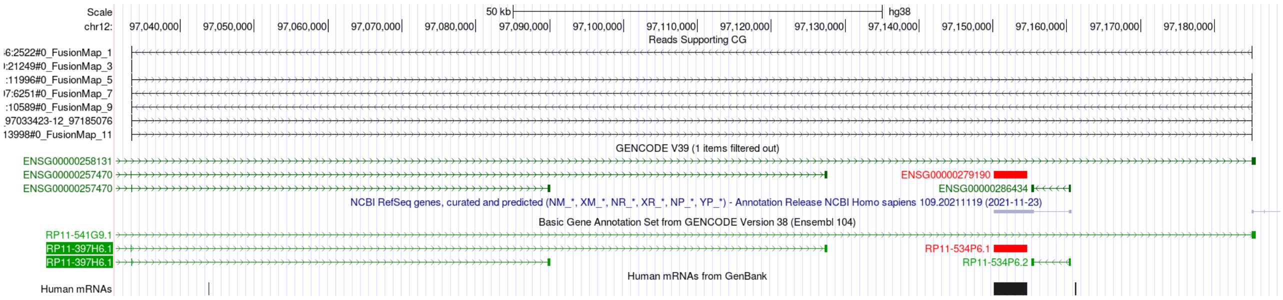

Supplementary Figure S11. (RP11-87G24.3::RP11-87G24.6)

Supporting reads for conjoined gene RP11-87G24.3::RP11-87G24.6. UCSC Genome Browser representation of the reads spanning over the two partners, as well as basic gene models and mRNA evidence are provided.

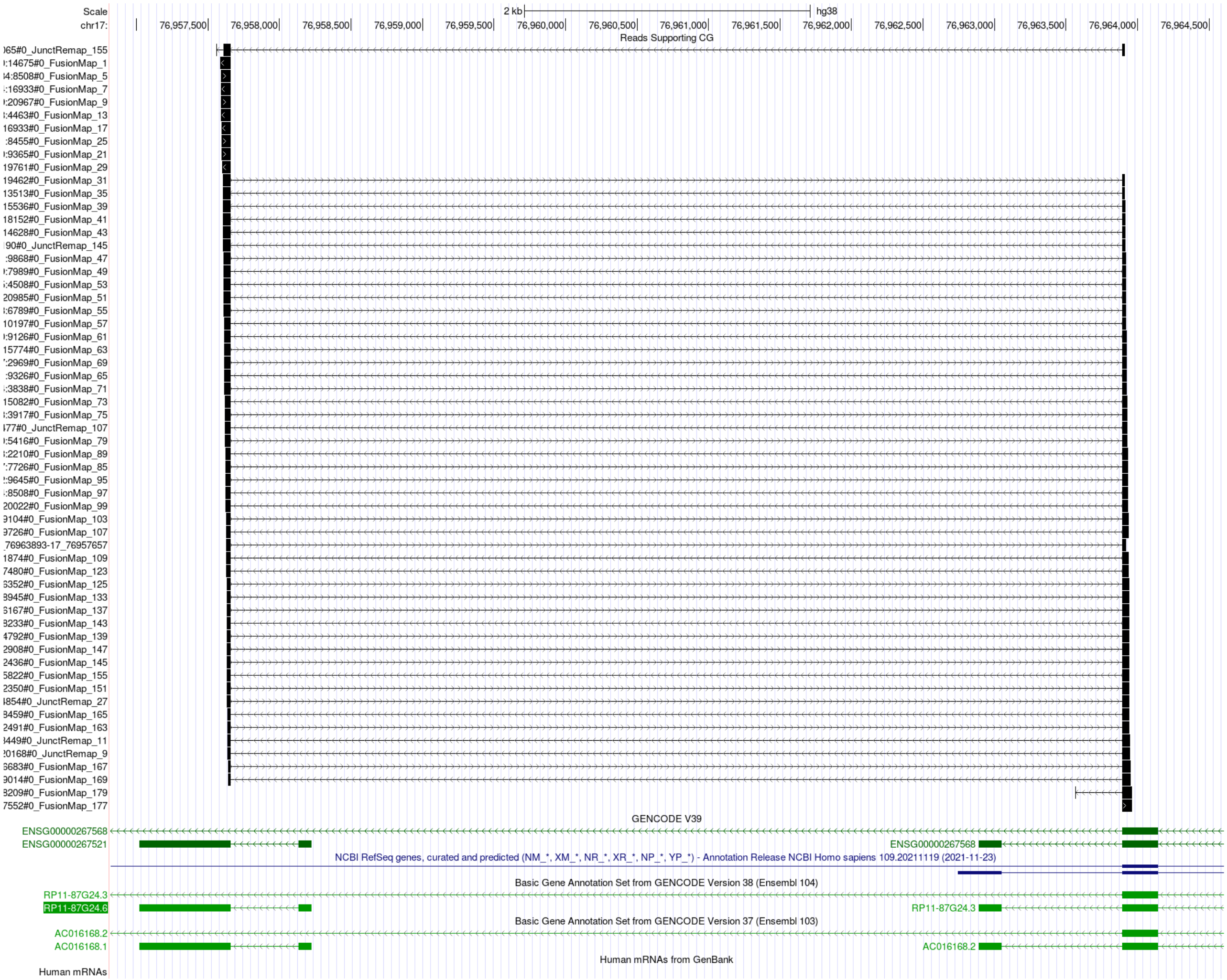

Supplementary Figure S12. (TMEM86A::RP11-1081L13.4)

Supporting reads for conjoined gene TMEM86A::RP11-1081L13.4. UCSC Genome Browser representation of the reads spanning over the two partners, as well as basic gene models and mRNA evidence are provided.

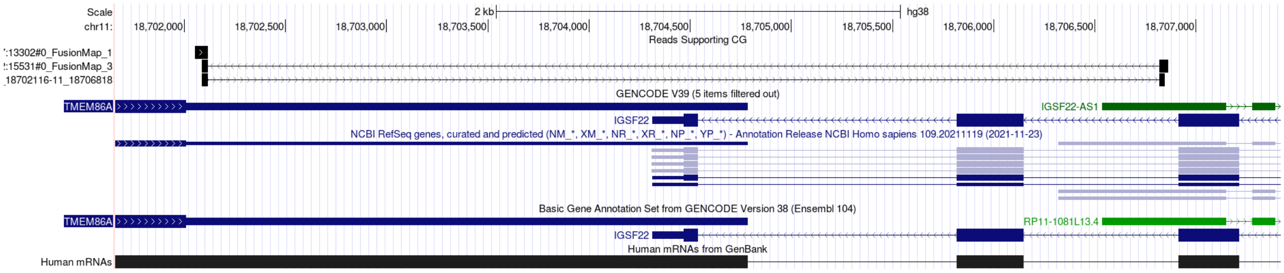

Fusion transcripts

Supplementary Figure S13. (DCAF8::ZNF836)

Supporting reads for fusion DCAF8::ZNF836. Integrated Genome Viewer (IGV) representation of the reads spanning over the two partners, as the fused sequence, the possible translation and the two gene partners are represented.

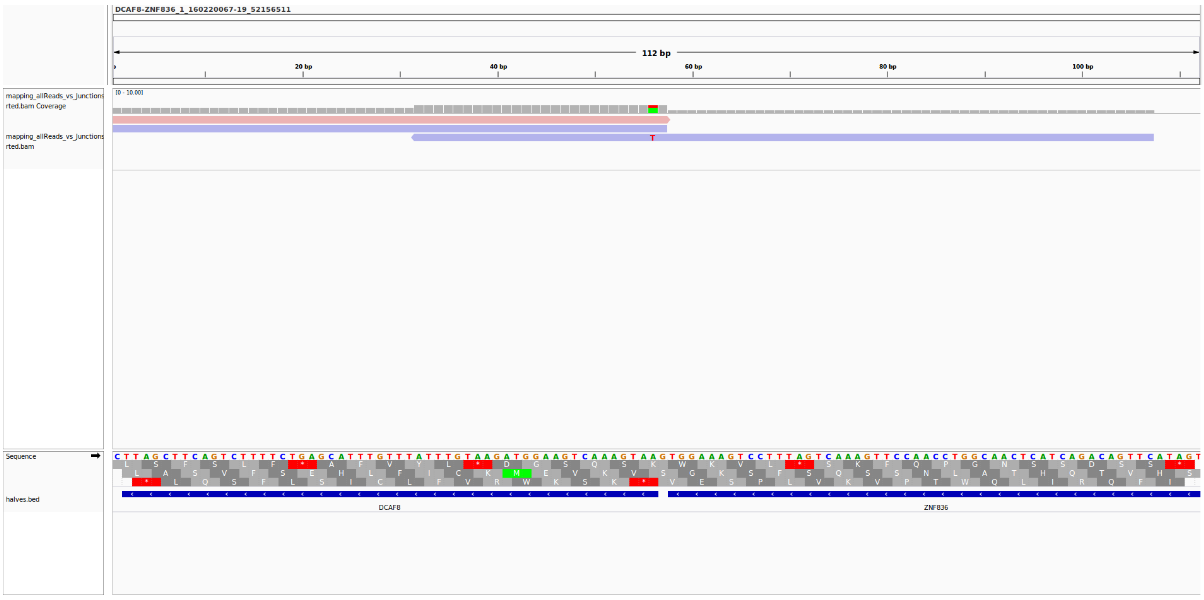

Supplementary Figure S14. (DMD::STAMBPL1)

Supporting reads for fusion DMD::STAMBPL1. Integrated Genome Viewer (IGV) representation of the reads spanning over the two partners, as the fused sequence, the possible translation and the two gene partners are represented.

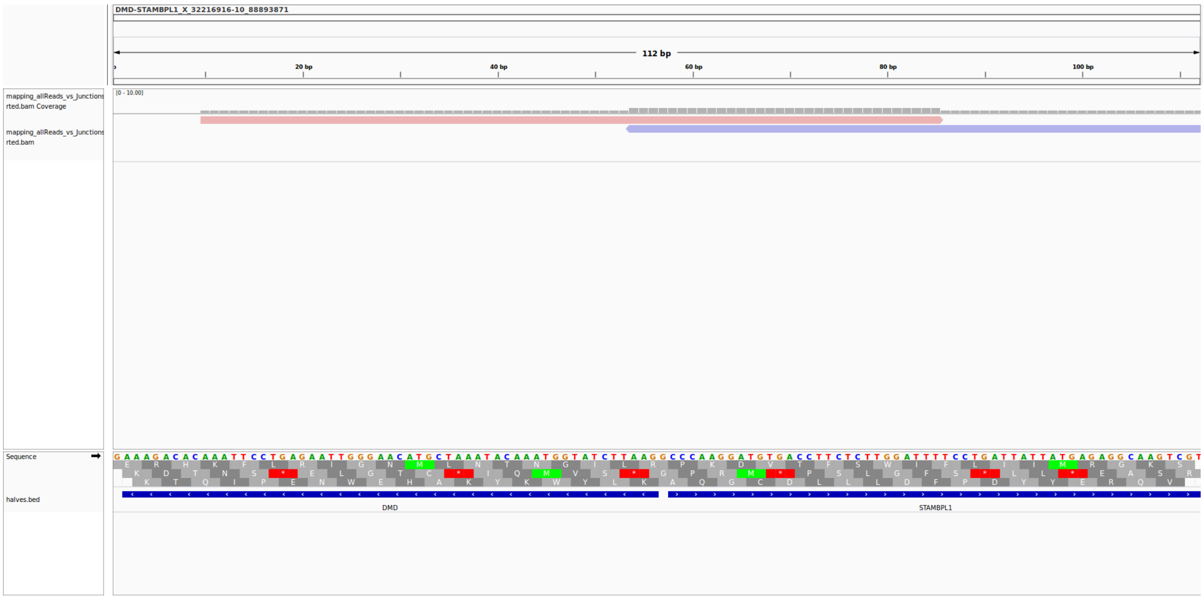

Supplementary Figure S15. (IK::FBXW2)

Supporting reads for fusion IK::FBXW2. Integrated Genome Viewer (IGV) representation of the reads spanning over the two partners, as the fused sequence, the possible translation and the two gene partners are represented.

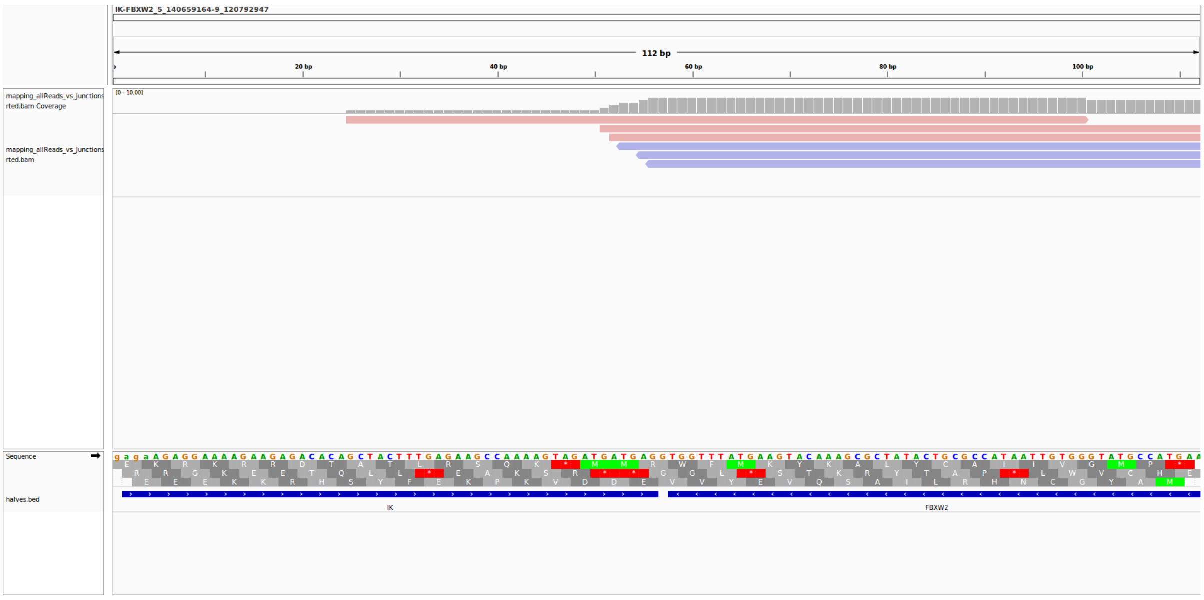

Supplementary Figure S16. (INPP5A::SETD7)

Supporting reads for fusion INPP5A::SETD7. Integrated Genome Viewer (IGV) representation of the reads spanning over the two partners, as the fused sequence, the possible translation and the two gene partners are represented.

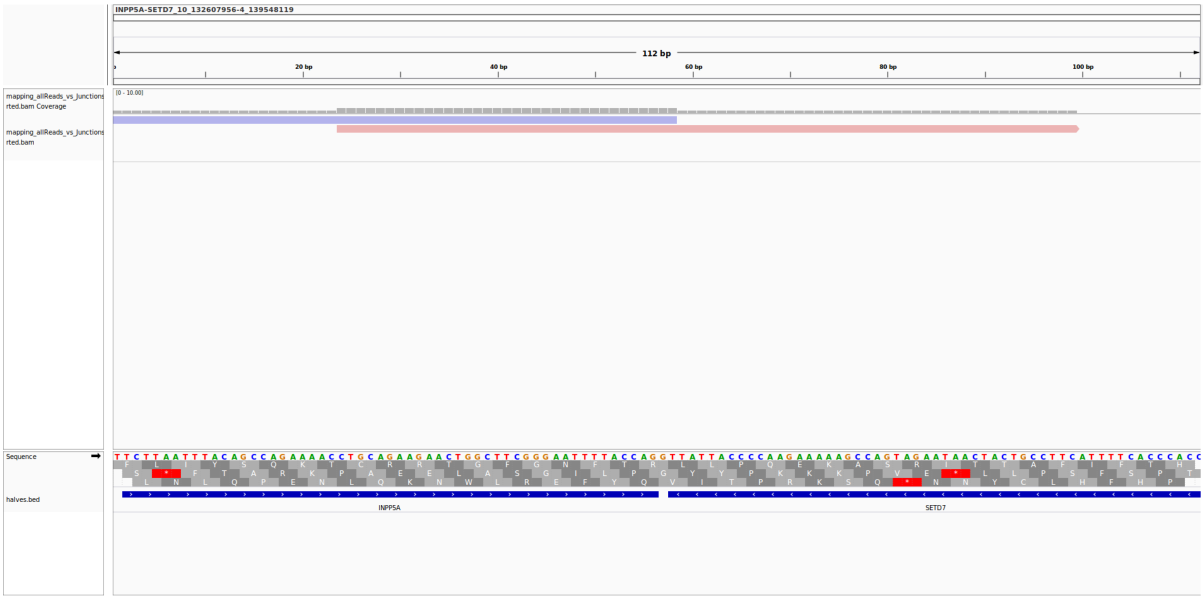

Supplementary Figure S17. (MAEA::CTBP1)

Supporting reads for fusion MAEA::CTBP1. Integrated Genome Viewer (IGV) representation of the reads spanning over the two partners, as the fused sequence, the possible translation and the two gene partners are represented.

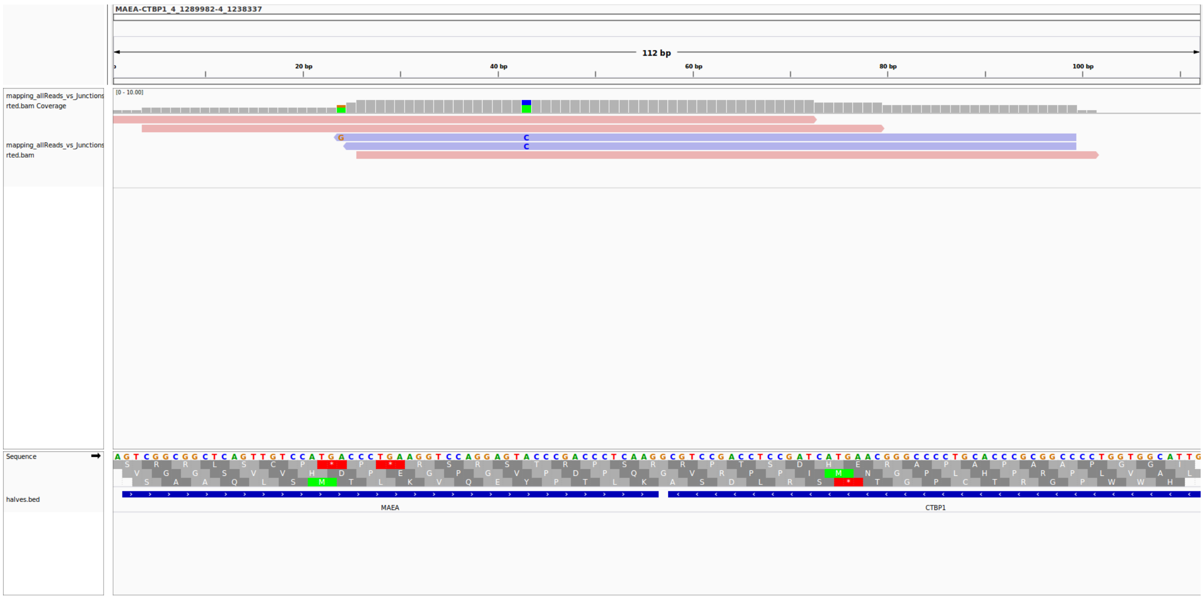

Supplementary Figure S18. (MAML2::FAT3)

Supporting reads for fusion MAML2::FAT3. Integrated Genome Viewer (IGV) representation of the reads spanning over the two partners, as the fused sequence, the possible translation and the two gene partners are represented.

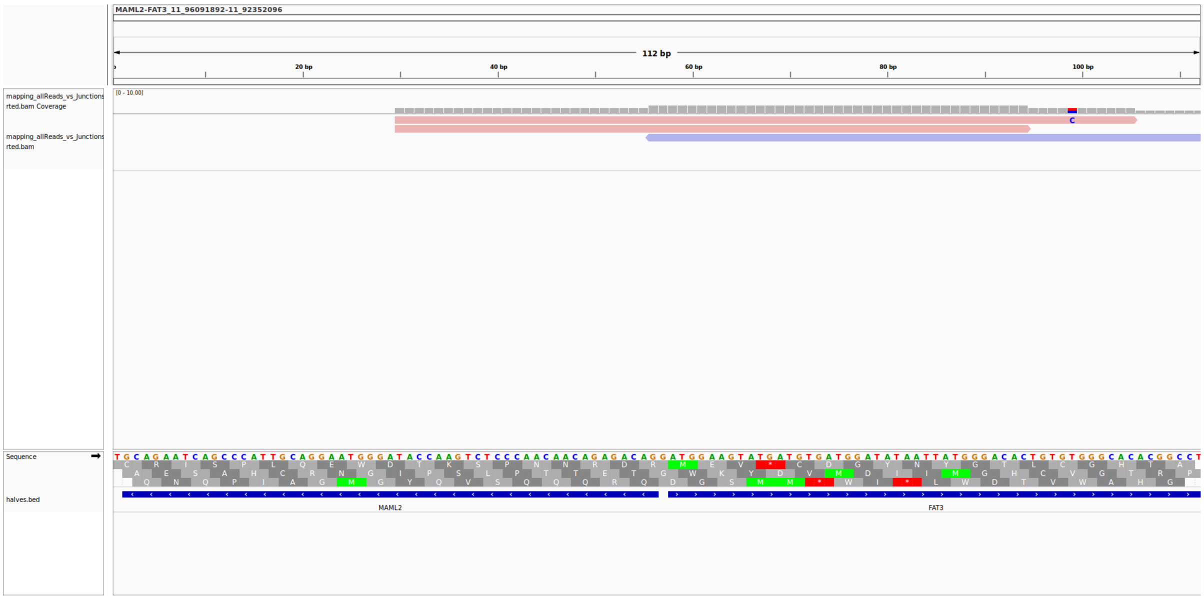

Supplementary Figure S19. (MNT::CLUH)

Supporting reads for fusion MNT::CLUH. Integrated Genome Viewer (IGV) representation of the reads spanning over the two partners, as the fused sequence, the possible translation and the two gene partners are represented.

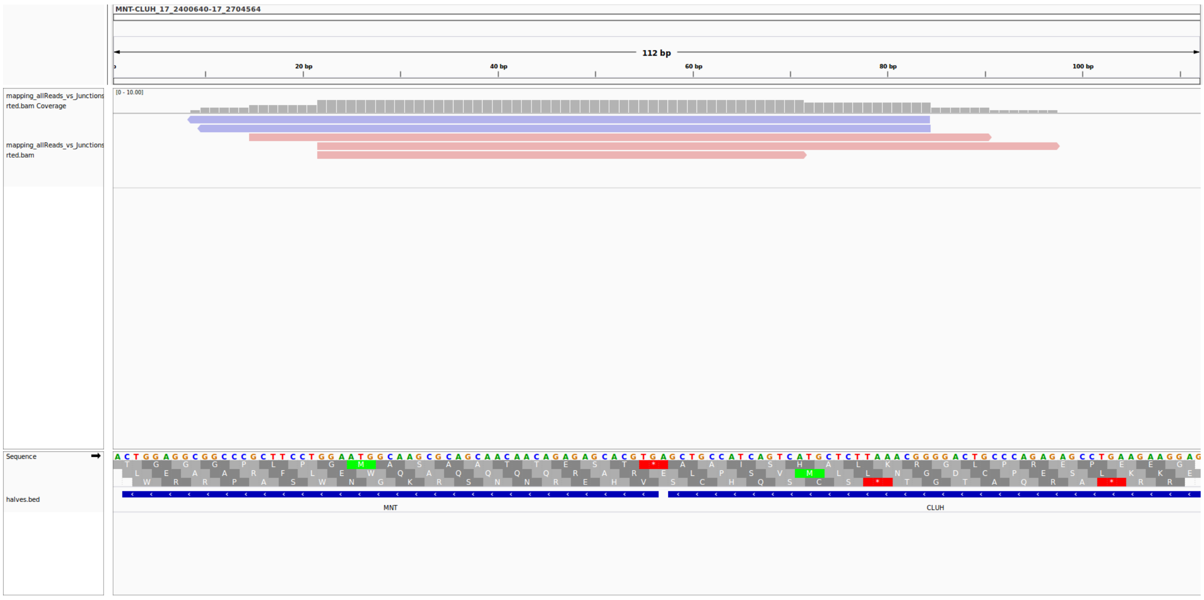

Supplementary Figure S20. (NFX1::DICER1)

Supporting reads for fusion NFX1::DICER1. Integrated Genome Viewer (IGV) representation of the reads spanning over the two partners, as the fused sequence, the possible translation and the two gene partners are represented.

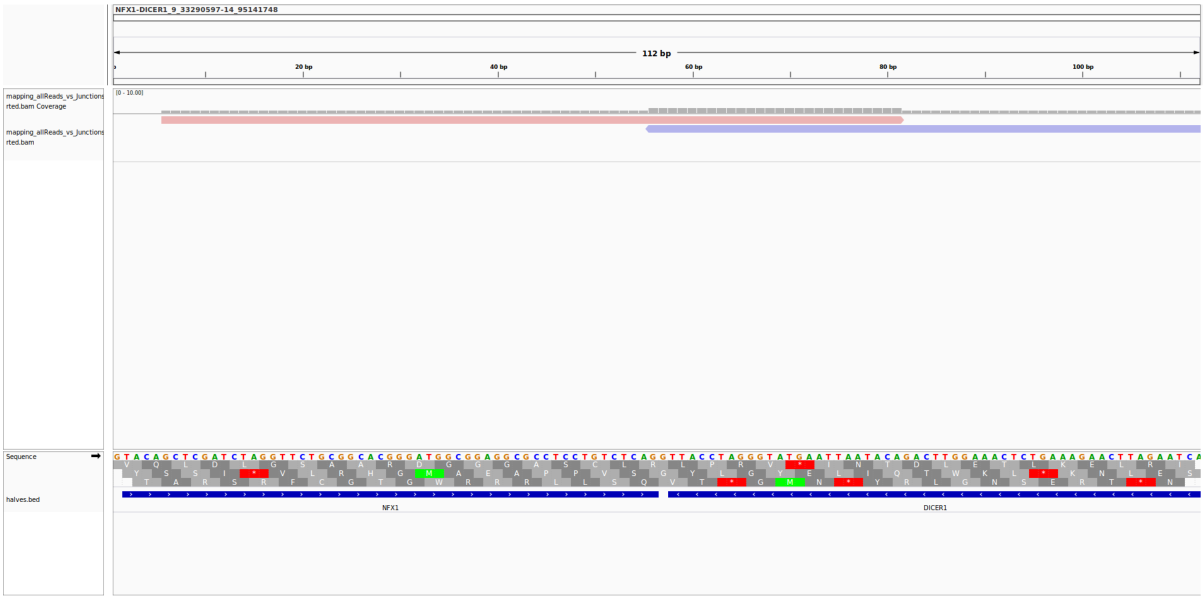

Supplementary Figure S21. (PAX5::POM121C)

Supporting reads for fusion PAX5::POM121C. Integrated Genome Viewer (IGV) representation of the reads spanning over the two partners, as the fused sequence, the possible translation and the two gene partners are represented.

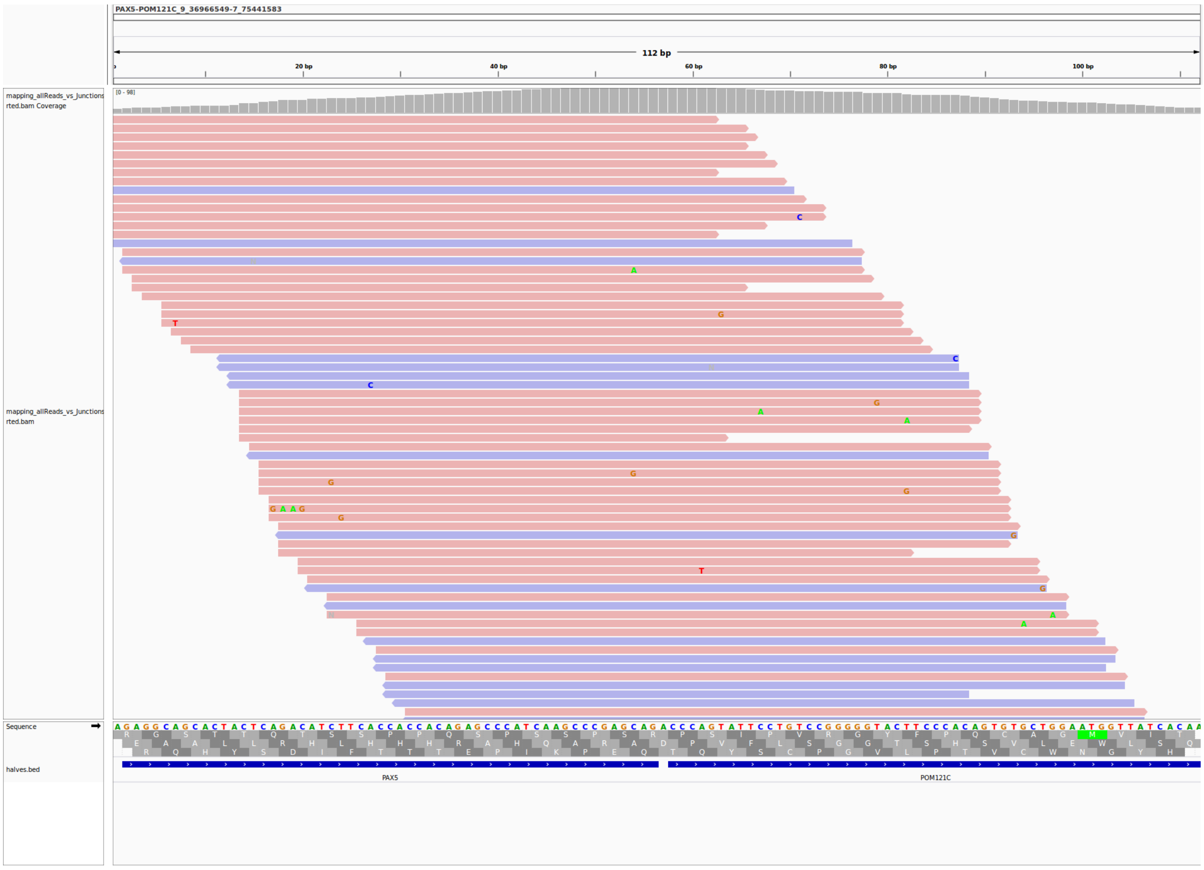

Supplementary Figure S22. (RP11-148O21.2::ATG4B)

Supporting reads for fusion RP11-148O21.2::ATG4B. Integrated Genome Viewer (IGV) representation of the reads spanning over the two partners, as the fused sequence, the possible translation and the two gene partners are represented.

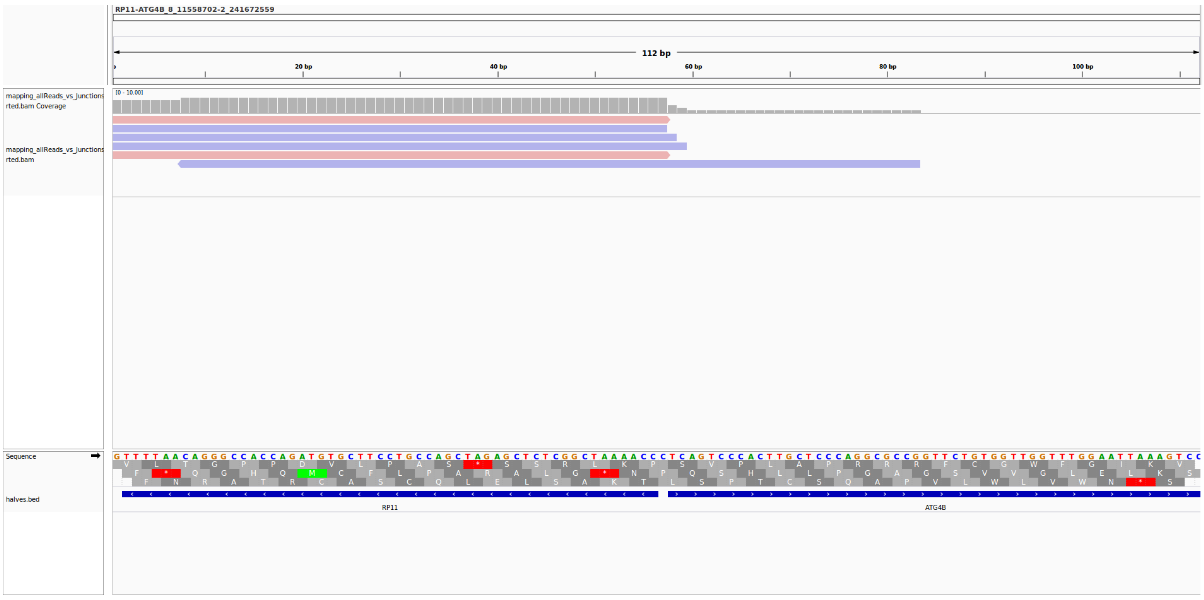

Supplementary Figure S23. (SLFNL1::SMPD2)

Supporting reads for fusion SLFNL1::SMPD2. Integrated Genome Viewer (IGV) representation of the reads spanning over the two partners, as the fused sequence, the possible translation and the two gene partners are represented.

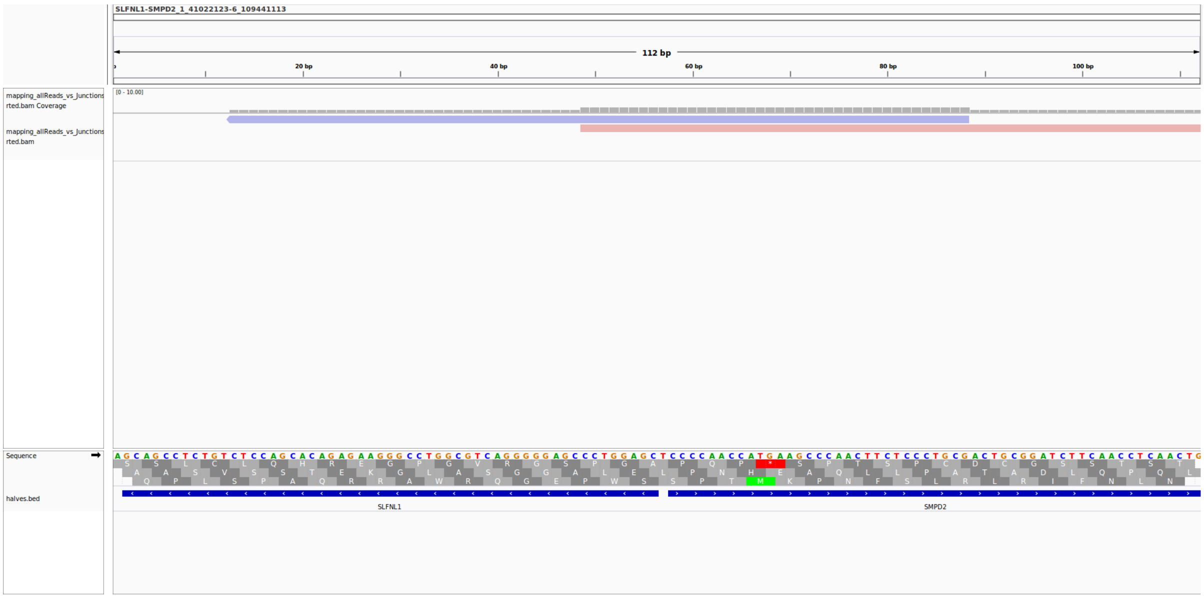

Supplementary Figure S24. (TMEM263::CD47)

Supporting reads for fusion TMEM263::CD47. Integrated Genome Viewer (IGV) representation of the reads spanning over the two partners, as the fused sequence, the possible translation and the two gene partners are represented.

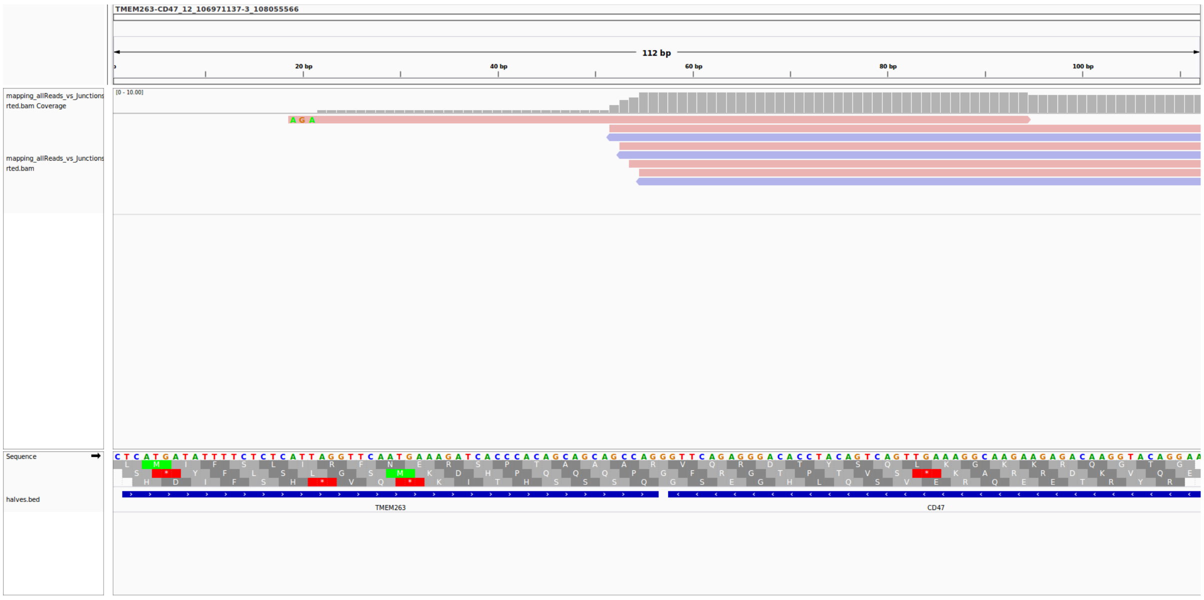
TMEM263-CD47_12_106971137-3_108055566
112 bp
20 bp
40 bp
60 bp
80 bp
100 bp
mapping_allReads_vs_Junctions rted.bam Coverage
[0 - 10.00]
mapping_allReads_vs_Junctions rted.bam
Sequence
halves.bed
TMEM263
CD47

Supplementary Figure S25. (TSKS::ARRDC2)

Supporting reads for fusion TSKS::ARRDC2. Integrated Genome Viewer (IGV) representation of the reads spanning over the two partners, as the fused sequence, the possible translation and the two gene partners are represented.

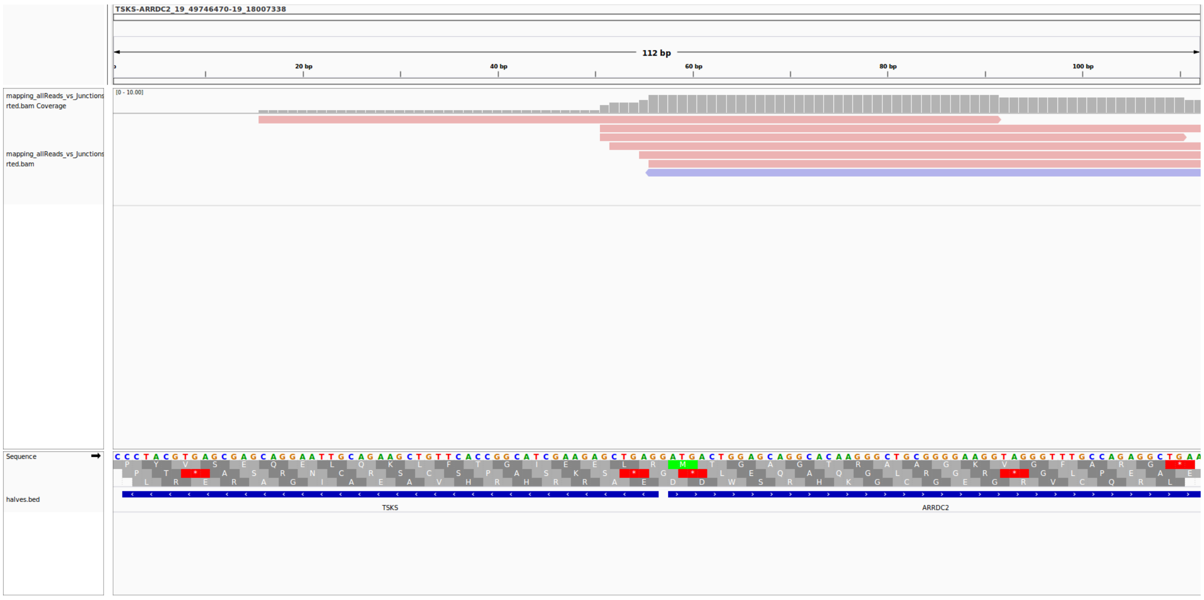

Supplementary Figure S26. (ZC3H12D::RP11-445F6.2)

Supporting reads for fusion ZC3H12D::RP11-445F6.2. Integrated Genome Viewer (IGV) representation of the reads spanning over the two partners, as the fused sequence, the possible translation and the two gene partners are represented.

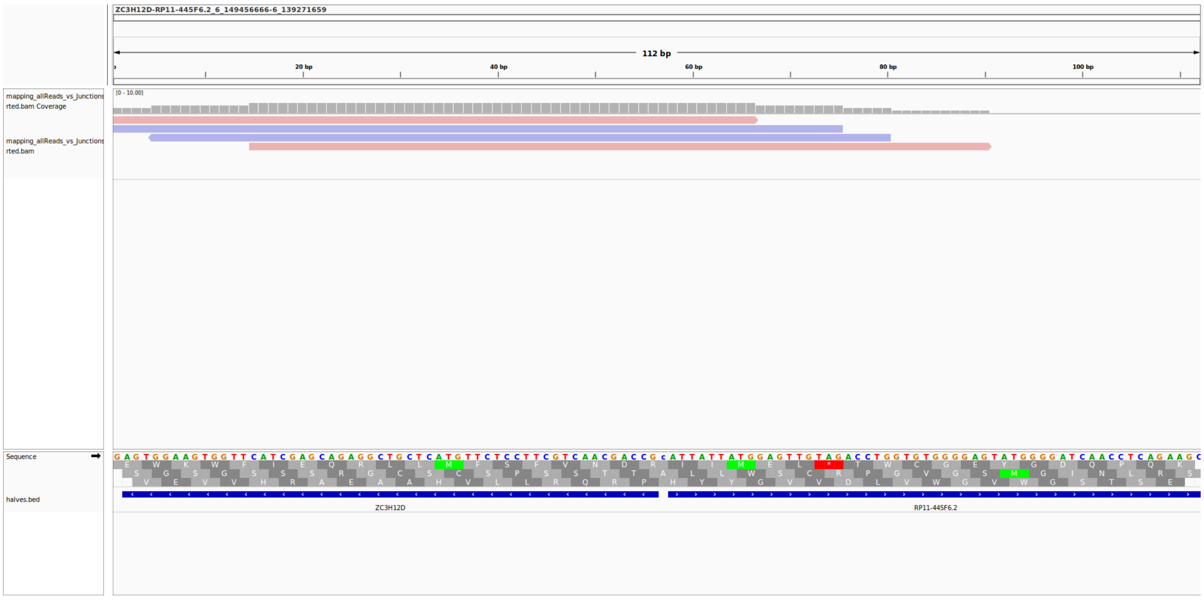

Supplementary Figure S27. (ZNF444::HLA-B)

Supporting reads for fusion ZNF444::HLA-B. Integrated Genome Viewer (IGV) representation of the reads spanning over the two partners, as the fused sequence, the possible translation and the two gene partners are represented.

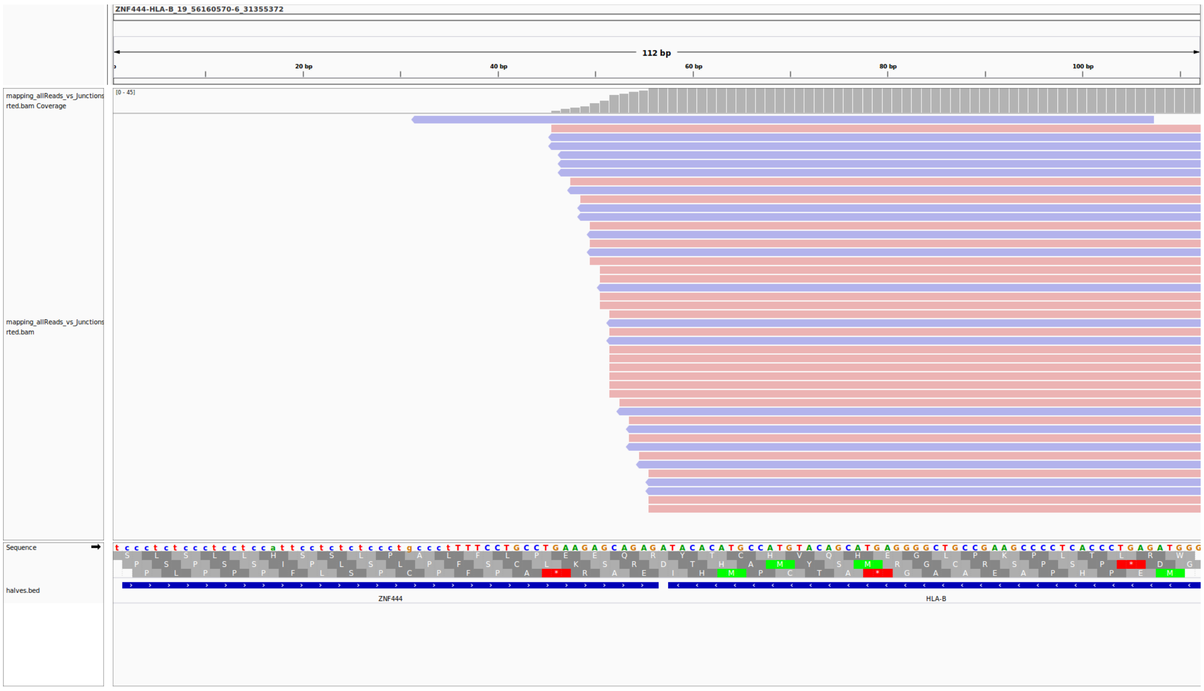

Supplement: Supplementary file 1 [file cancers-14-03523-s001.zip › supplementary materials/Supplementary Figures.pdf]
